# Biosynthetic gene cluster profiling from North Java Sea *Virgibacillus salarius* reveals hidden potential metabolites

**DOI:** 10.1038/s41598-023-44603-8

**Published:** 2023-11-06

**Authors:** Ocky Karna Radjasa, Ray Steven, Zalfa Humaira, Fenny Martha Dwivany, Husna Nugrahapraja, Joko Pebrianto Trinugroho, Tati Kristianti, Agus Chahyadi, Yosua Natanael, Neil Priharto, Kamarisima, Farisa Amalia P. B. Sembiring, Ari Dwijayanti, Lia Kusmita, Maelita R. Moeis, V. Sri Harjati Suhardi

**Affiliations:** 1 Research Center for Deep Sea, The Earth Sciences and Maritime Research Organization, National Research and Innovation Agency, Jakarta, 14430 Indonesia; 2 Institut Teknologi Bandung, School of Life Sciences and Technology, Bandung, West Java 40132 Indonesia; 3 Institut Pendidikan Indonesia, Garut, West Java 44151 Indonesia; 4 University Center of Excellence for Nutraceuticals, Bioscience and Biotechnology Research Center, Bandung Institute of Technology, Bandung, West Java Indonesia; 5 CNRS@CREATE Ltd., 1 Create Way, #08-01 Create Tower, Singapore, 138602 Singapore; 6 STIFAR Yayasan Pharmasi Semarang, Semarang, Central Java 50124 Indonesia; 7 Department of Biotechnology, Faculty of Science and Technology, Universitas Muhammadiyah Bandung, Bandung, West Java 40262 Indonesia

**Keywords:** Biotechnology, Computational biology and bioinformatics, Molecular biology

## Abstract

*Virgibacillus salarius 19.PP.SC1.6* is a coral symbiont isolated from Indonesia's North Java Sea; it has the ability to produce secondary metabolites that provide survival advantages and biological functions, such as ectoine, which is synthesized by an ectoine gene cluster. Apart from being an osmoprotectant for bacteria, ectoine is also known as a chemical chaperone with numerous biological activities such as maintaining protein stability, which makes ectoine in high demand in the market industry and makes it beneficial to investigate *V. salarius* ectoine. However, there has been no research on genome-based secondary metabolite and ectoine gene cluster characterization from Indonesian marine *V. salarius*. In this study, we performed a genomic analysis and ectoine identification of *V. salarius*. A high-quality draft genome with total size of 4.45 Mb and 4426 coding sequence (CDS) was characterized and then mapped into the Cluster of Orthologous Groups (COG) category. The genus *Virgibacillus* has an "open" pangenome type with total of 18 genomic islands inside the *V. salarius* 19.PP.SC1.6 genome. There were seven clusters of secondary metabolite-producing genes found, with a total of 80 genes classified as NRPS, PKS (type III), terpenes, and ectoine biosynthetic related genes. The ectoine gene cluster forms one operon consists of *ectABC* gene with 2190 bp gene cluster length, and is successfully characterized. The presence of ectoine in *V. salarius* was confirmed using UPLC-MS/MS operated in Multiple Reaction Monitoring (MRM) mode, which indicates that *V. salarius* has an intact ectoine gene clusters and is capable of producing ectoine as compatible solutes.

## Introduction

The sea constitutes a dynamic and intricate ecosystem. Physical factors such as atmospheric pressure and temperature, as well as chemical factors like pH and salt concentration, exhibit variations at various ocean depths. These abiotic fluctuations significantly impact marine microorganisms, compelling them to adapt and evolve to thrive in these challenging surroundings. Among the adaptation mechanisms observed in marine microorganisms is the synthesis of a wide range of secondary metabolites^1, 2^.

Secondary metabolites exhibit a wide range of biochemical activities, encompassing antibiotics, antioxidants, and antitumor properties^3, 4^. These compounds are typically generated by Secondary Metabolite Biosynthetic Gene Clusters (smBGCs), which are sets of genes that govern specific metabolic pathways responsible for secondary metabolite production^5^. In recent times, secondary metabolites possessing antimicrobial properties have found application in antibiotic production within the pharmaceutical industry, garnering approval from the United States Food and Drug Administration (FDA) agencies^6^. Secondary metabolites sourced from marine environments boast distinctive and diverse chemical structures, piquing the pharmaceutical industry's interest, particularly for the potential discovery of novel antibiotic compounds^7^. Marine-derived secondary metabolites have found utility across various sectors, including agriculture as biopesticides^8^, the food industry as functional additives^9^, and the cosmetics and skincare sector as ingredients for beauty products^10^.

Genome mining has emerged as an indispensable tool for tapping into the vast potential of Indonesia's deep sea and its valuable biological compounds. Indonesia, renowned as the world's largest archipelagic nation and one of the most biodiverse, boasts an expansive oceanic territory that covers a remarkable 77% of its total land area, with sea depths exceeding 200 meters^11^. This phenomenon is particularly prominent in Eastern Indonesia, encompassing regions like the Flores Sea, Banda Sea, and Makassar Strait, as well as the open waters of the Indian and Pacific Oceans^12^. As a result, Indonesia offers an extraordinary opportunity for the discovery of valuable biological compounds.

Conventional biochemical methods used for the screening and isolation of secondary metabolites from bacterial species have inherent limitations. These methods demand extensive labor and entail the manipulation of various cultivation parameters, including light, pH, aeration, temperature, and nutrient composition^13^. Furthermore, a significant challenge arises from the fact that numerous biosynthetic gene clusters (BGCs) responsible for secondary metabolite production remain silent or cryptic under standard laboratory conditions. These gene clusters are predominantly activated in response to environmental stressors, driving bacterial adaptations^14^.

To surmount these limitations, genome mining emerges as a promising alternative. This method harnesses the power of genome sequencing, computational analysis, and compound characterization, enabling the discovery of novel natural bioproducts^15, 16^. Genome mining has demonstrated its efficacy in uncovering the potential of marine microbial compounds that have eluded detection using traditional characterization methods, including cultivation and isolation within laboratory settings^5, 17, 18^.

One of the secondary metabolites produced by certain marine bacteria to serve as an osmoprotectant is ectoine. Ectoine facilitates microbial adaptation to fluctuations in salinity within the sea, employing a "salt-out" strategy^19^. Ectoine, chemically known as 1,4,5,6-tetrahydro-2-methyl-4-pyrimidine carboxylic acid, is an amino acid derivative originating from aspartate, possessing a pronounced affinity for water^20^. The synthesis of ectoine involves three principal enzymes encoded by three genes: *ectA*, *ectB*, and *ectC*, which collectively form a gene cluster. Previous research has demonstrated the conservation of ectoine biosynthetic gene clusters across numerous bacterial species^21^. Traditionally, the production of ectoine has relied on the use of *Halomonas elongata*, a halophilic bacterium. The process involves stimulating these bacteria within a high-salinity medium, followed by inducing an osmotic shock to release ectoine into the surrounding medium^22^.

**Figure 1:**
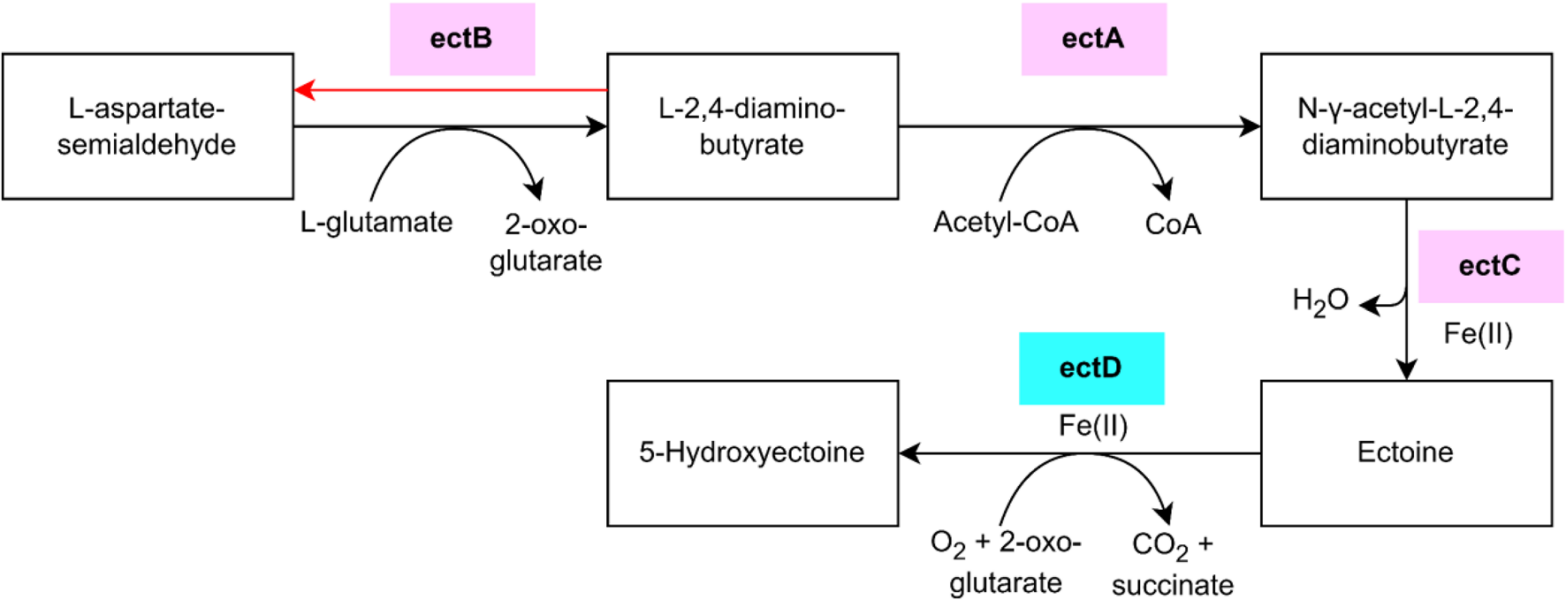
Ectoine and 5-hydroxyectoine biosynthesis pathway. This pathway consists of *ectA*, *ectB*, and *ectC* as main genes. Not every ectoine-producing bacteria has the *ectD* gene, whose main activity is to produce ectoine derivative 5-hydroxyectoine.

Ectoine, often referred to as a chemical chaperone, plays a crucial role in stabilizing proteins, shielding against UV radiation, and safeguarding molecules or cells from high temperatures. Its versatile properties make it a potential natural protectant with applications spanning agriculture, biotechnology, healthcare, food processing, and cosmetics^22^. In the realm of skincare, ectoine finds extensive use as an ingredient, contributing significantly to sun protection and anti-aging products. It also serves as an enhancer of lipase catalytic efficiency in biodiesel production and is utilized in the healthcare industry to develop ectoine-based medical devices for allergy treatment, including nasal sprays and eye drops^22–24^. The biosynthesis of ectoine commences with the presence of a precursor called L-aspartate-semialdehyde, which undergoes conversion to L-2,4-diaminobutyrate through the action of L-2,4-diaminobutyrate-2-oxoglutarate transaminase (*ectB*). Subsequently, diaminobutyric acid acetyltransferase (*ectA*) transforms it into N-γ-acetyl-L-2,4-diaminobutyrate, ultimately leading to the production of ectoine, facilitated by ectoine synthase (*ectC*)^21^. In the presence of the *ectD* gene, which encodes ectoine hydroxylase, certain bacteria, such as *V. halodenitrificans* and *V. salarius*, can produce ectoine derivatives in the form of 5-hydroxyectoine^23^ (Fig. 1).

Considering the relatively uncharted territory of Indonesia's marine bioresources, there is a pressing need for further exploration through a genomics-driven strategy. This approach holds the promise of unearthing previously unknown and potentially valuable bioproducts, including bacteria such as *V. salarius*. Industries are actively seeking cost-effective methods for a variety of bioproducts, and the discovery of a bacterial strain with unique capabilities would be of great interest. While *V. salarius* 19.PP.SC1.6, isolated from coral in the North Java Sea, has shown potential in the synthesis of various industrially valuable compounds^25–28^, its complete genome sequence and specific capabilities remain uncharted territory. Conducting comprehensive research to obtain genomic data on Indonesia's marine microbes, including *V. salarius* 19.PP.SC1.6, has the potential to unlock the vast capabilities of these natural resources and their application across various industries. This knowledge will significantly contribute to the development of sustainable and economically viable processes for the production of valuable compounds. Further investigation is essential to delineate the specific bioproduct capabilities of *V. salarius* 19.PP.SC1.6 and assess its potential as a high-yield ectoine producer.

## Material and methods

### Sample collection and cultivation

The bacterial samples utilized in this study were sourced from our prior studies^28^. Specifically, soft coral *Sinularia* sp. specimens were collected at a depth of 2 m in the waters of Pulau Panjang, Jepara, Central Java (coordinates: 6°34′37.35ʺLS 110°37′52.01ʺE and 6°34′41.38ʺLS 110°37′54.12ʺE). These coral specimens measured 3 to 5 cm in size and were placed in a plastic container filled with sterile seawater before being temporarily stored in a coolbox. The symbiotic microorganisms were isolated following the method outlined by Kusmita et al.^28^.

### Isolation and sequencing of genomic DNA

Bacterial DNA isolation was carried out using the PowerWater® DNA Isolation Kit from MOBIO, while Whole Genome Amplification (WGA) was performed using the REPLI-G Mini Kit from QIAGEN (Venlo, Netherlands). Subsequently, the DNA sample underwent whole genome sequencing, which was conducted by Genetika Science Indonesia (Jakarta, Indonesia), utilizing the GridION sequencing platform from Oxford Nanopore Technologies (Oxford, U.K).

### Identification and taxonomic analysis

The Mash algorithm (MinHash) was employed on the web PATRIC 3.6.12^29^ platform with a p-value of 0 to search for related species. GenBank files generated from whole genome sequencing were parsed using a Python script (available at https://github.com/raysteven/gbk_parser), which was executed using Biopython 1.79. ANI (Average Nucleotide Identity) values were computed using FastANI version 0.1.3^30^, while dDDH (digital DNA-DNA hybridization) values were calculated using GGDC (Genome-to-Genome Distance Calculator) version 3.0^31^. To construct a phylogenomic tree from the dataset, OrthoFinder version 2.5.4^32^ was utilized, and the resulting tree was visualized with iTOL version 6.5.2^33^. The genome assembly of *V. salarius* 19.PP.SC1.6 has been deposited in the NCBI GenBank database under the accession number GCF_027941815.1.

### Estimation of genomic characteristics

The total genome length (in base pairs, bp), coding region length (in bp), total G+C content (%), and the number of genes (including tRNA, rRNA, tmRNA, and CDS) were computed using the Python script mentioned (https://github.com/raysteven/gbk_parser). Subsequently, the obtained results were visualized as a circular genome map using the Proksee web server's CGView-based draw engine^34^.

### Pan genome, genomic islands, and secondary metabolites analysis

For pan genome analysis, GET HOMOLOGUES^35^ was utilized to acquire occupancy statistics, estimate the size of the pangenome and core genome, and perform related analyses. To predict the presence of genomic islands, the IslandViewer web server version 4 was employed^36^. The antiSMASH web server version 6.0.1 was used to predict the existence of biosynthetic gene clusters responsible for secondary metabolite production^37^. Visualization of reaction pathways, based on KO (KEGG Orthology) annotations, was conducted using the KEGG Reconstructor^38^.

### In silico nonribosomal peptides structure similarity search and its antibacterial activity prediction

The putative non-ribosomal peptides (NRP) chemical structures were extracted from the antiSMASH results in SMILES (simplified molecular-input line-entry system) format. These NRP SMILES were then employed as search queries in the NRP database, using NORINE (https://bioinfo.lifl.fr/norine/), to identify potential structural similarities with known NRP compounds existing in the database^39^. Additionally, the NRP SMILES were utilized to predict the in silico antibacterial effects through QSAR (quantitative-structure activity relationship) methods using the antiBac-Pred web application (http://www.way2drug.com/antibac/) from Way2Drug^40^.

### Ectoine biosynthetic gene cluster characterization and comparison

Comparison of *V. salarius* 19.PP.SC1.6 ectoine gene sequences and clusters with ectoine gene cluster sequences obtained from the NCBI database, which have accession numbers AY935521 (*Virgibacillus salexigens* DSM 11483), KU510274 (*Virgibacillus halodenitrificans* PDB-F2), AY585263 (*Virgibacillus pantothenticus* DSM 26), DQ471210 (*Bacillus alcalophilus* DTY1), MH020162 (*Bacillus clausii* NIOT-DSB04), U66614 (*Marinococcus halophilus* DSM 20408), and D88359 (*Halomonas elongata* OUT30018), was carried out through multiple sequence alignment (MSA). A Neighbor Joining (NJ) phylogenetic tree was constructed using MEGA-X software^41^ for further analysis of identity and sequence similarity using SIAS^42^. The results were visualized using IBS^43^. Structural features of the ectoine gene cluster were predicted using various tools, including the Promoter Prediction tool (http://www.fruitfly.org/seq_tools/promoter.html)^44^, BPROM^45^, and FindTerm^45^. These tools were employed to identify promoter regions and termination sites within the ectoine gene cluster of *V. salarius* 19.PP.SC1.6.)

### Ectoine production and identification

*V. salarius* 19.PP.SC1.6 was cultured in 100 mL of Zobell Marine Medium (Himedia M384-500G) containing a 10% NaCl concentration, with agitation at 150 rpm for 30, 45, and 48 h. To collect cell pellets, the culture was subjected to centrifugation at 6000 rpm for 10 min. Following this, 100 mL of 1.5% NaCl was added for the bacterial milking process. Bacterial milking was performed at 200 rpm and 25 °C for a duration of 30 min. The resulting cell suspension was then centrifuged at 8000 rpm for 15 min. The supernatants obtained from the bacterial milking process were subsequently freeze-dried for further analysis.

### Ectoine analysis using liquid chromatography-mass spectrometry (LC–MS)

The authentic standard of ectoine obtained from Sigma Aldrich (Missouri, USA) was freshly prepared in distilled water and sonicated for 2 min. A stock solution of 1 mg/ml concentration was prepared and then filtered through a 0.22-µm PTFE filter for analysis. The analysis was conducted using an Acquity UPLC H-Class system equipped with a binary solvent manager, sample manager, and column heater, all from Waters (Michigan, USA). The Masslynx analysis software was employed to operate the instrument and carry out data analyses. A separation column, ACQUITY UPLC BEH Shield RP18 100 × 2.1 mm with 1.7-μm particle size, was utilized. The mobile phase consisted of two solvents: solvent A, which was composed of water with 0.1% formic acid, and solvent B, which contained acetonitrile (AcN) with 0.1% formic acid. The elution was performed in isocratic mode using 5% B for 10 min at a flow rate of 0.3 mL/min. The column was maintained at a temperature of 40 °C, and the injection volume for both the sample and standard was 3 µL.

Detection was carried out using multiple reaction monitoring (MRM), a highly specific and sensitive mass spectrometry (MS) technique that can selectively quantify compounds within complex mixtures by monitoring multiple daughter ions of the compound of interest. Electrospray ionization (ESI) was operated in positive mode (ESI +) with the following source parameters: a spray voltage of 26 V using nitrogen as the source gas, a source gas flow rate of 900 L/h for desolvation, and a source temperature of 450 °C for desolvation. The collision energies were set to 15 V for the quantifier and 25 V for the qualifier. Capillary voltage and cone voltage were maintained at 2.5 kV and 26 V, respectively. Under these conditions, product ions (m/z) for ectoine were observed as follows: 143.02 for the parent ion, 97.21 for the quantifier, and 68.21 for the qualifier.

### Ectoine genes isolation

*V. salarius* 19.PP.SC1.6 underwent a cultivation process, initially on solid Zobell Marine Broth Medium, followed by subculturing in liquid Zobell Marine Medium, with overnight incubation at 37 °C in a shaker set at 200 rpm. DNA isolation was performed using the ZymoBIOMICS DNA Miniprep Kit from Zymo Research (Irvine, California), in accordance with the provided protocol. The *ectABC* biosynthetic gene was amplified through PCR, with denaturation temperatures at 95 °C, annealing temperatures at 53 °C, and elongation temperatures at 72 °C. Specific primers were utilized for this purpose: *ectA* primer (5′-atgcctacaaaagatgatggggcag-3′ F and 3′-ttattcattatttcccttttgaaaagggccaatctt-5′ R), *ectB* primer (5′-atgaaaacctttgaagaattggaatcatcggt-3′ F and 3′-ttacttcaatacttgtttaattgcttcttcaagaa-5′ R), and *ectC* (5′-atgatcgtaaaatcacttgaagatattattggaac-3′ F and 3′-ttattctgttaatagaggataatatccttcttttg-5′ R). Subsequently, the amplified gene was purified using the Wizard® SV Gel and PCR Clean-Up System kit.

For gene cloning, the *ectA*, *ectB*, and *ectC* genes were ligated into the pGEM®-T Easy Vector plasmid obtained from Promega (Wisconsin, USA) following established protocols. Transformation of *E. coli* DH5α with plasmids containing these genes was accomplished using the heat shock method^46^, and the transformed bacteria were cultured on ampicillin LB medium for selection. The cloning vector containing the *ectA*, *ectB*, and *ectC* genes was isolated from the recombinant *E. coli* DH5α and verified through PCR using gene-specific primers. Finally, the recombinant plasmid was confirmed through plasmid sequencing to ensure the successful integration of the *ectA*, *ectB*, and *ectC* genes.

## Results and discussion

### Environmental conditions

*V. salarius* 19.PP.SC1.6 was isolated from Panjang Island in the North Java Sea, where it was found to inhabit an environment with a salinity level of approximately 2.6%, as indicated in Table 1. This bacterium belongs to the category of moderate halophilic bacteria, which means it can thrive in a wide range of environmental conditions. It is capable of living in salt concentrations ranging from 0.5% to 25%, accommodating pH levels between 5.5 and 10, and tolerating temperatures within the range of 10 °C to 50 °C^46^. The successful isolation of this bacterium from its natural habitat aligns with its ecological profile, which falls well within its documented growth parameters^46^.

**Table 1:** Descriptive information on the environmental characteristics of Panjang island (sampling location)^28^.

Parameter	Panjang island
Salinity (‰)	26
pH	7.08
Temperature (℃)	28.8
Visibility (m)	1
DO (mg/L)	3.45
NH_3_ (mg/L)	2.5
S^2−^ (mg/L)	0.000
NO_3_ (mg/L)	2.6

### Identification and taxonomic analysis

**Figure 2:**
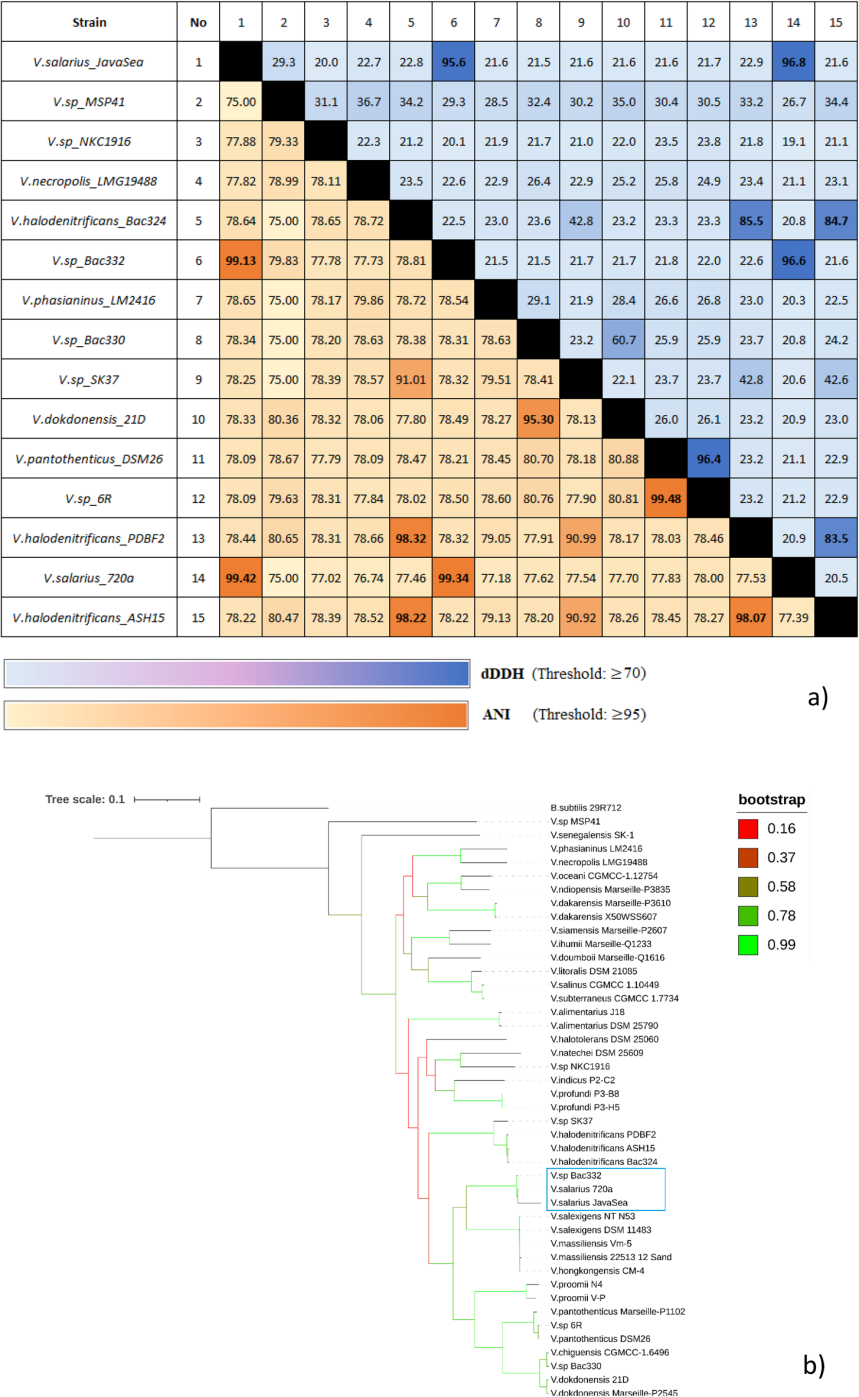
(**a**) Calculation results of ANI and dDDH of 14 *Virgibacillus* genomes for sample identification. (**b**). Phylogenomic analysis of the *V. salarius* 19.PP.SC1.6 genome compared to 43 other *Virgibacillus* genomes in total. Phylogenomic tree was constructed using OrthoFinder and Visualized with iTOL software.

The Overall Genome Relatedness Index (OGRI), which encompasses ANI (%) and digital DNA-DNA Hybridization (dDDH) (%), serves as a tool for identifying bacterial samples collected, as depicted in Fig. 2a. The calculated OGRI values for *V. salarius* 19.PP.SC1.6 indicate ANI (99.42%) and dDDH (96.8%) when compared to *V. salarius* 720a. Notably, the ANI threshold value is set at 95%^47^, and the dDDH^48^ threshold value is set at 70%. These set threshold values are used to determine whether two genomes belong to the same species. These results strongly suggest that *V. salarius* 19.PP.SC1.6 and *V. salarius* 720a belong to the same species. To further bolster this identification, a phylogenomic tree was constructed (Fig. 2b), which solidifies *V. salarius* 19.PP.SC1.6 as the closest relative to *V. salarius* 720a within this species tree. These findings corroborate the conclusions drawn from the previous 16S rRNA analysis, reaffirming the bacterial identity as *V. salarius*^28^.

### Genomic characteristics

Table 2 presents the general characteristics of the *V. salarius* 19.PP.SC1.6 genome. The genome size of *V. salarius* 19.PP.SC1.6 is approximately 4.45 Mbp. It's worth noting that bacteria with genome sizes larger than 3 Mbp often exhibit a higher G+C content. This phenomenon is attributed to multiple horizontal gene transfer (HGT) events involving various genomic elements, including genomic islands, which occur in response to environmental pressures, ultimately enhancing their survival strategies^49^. The differences observed among the numbers of COG, KO, COG-KO-annotated, and unannotated CDS indicate that there are many CDSs that remain insufficiently characterized, underscoring the need for further research.

**Table 2:** General characteristics of the *V. salarius* 19.PP.SC1.6 genome from whole genome sequencing (WGS) result.

Attribute	Score	% from total*
Genome size (bp)	4,497,283	100.00
DNA coding region (bp)	3,575,955	79.51
DNA G+C content (bp)	1,650,503	36.70
Total genes	4979	100.00
rRNA	17	0.34
tRNA	64	1.29
tmRNA	1	0.02
CDS	4897	98.35
CDS annotated with COG	4426	88.89
CDS annotated with KO	4297	87.75
CDS annotated with COG & KO	4227	84.90

*Total is from genome size (bp) or from total genes.

*COG* Clusters of Orthologous Groups, *CDS* Coding Sequence, *KO* KEGG Orthology.

**Figure 3:**
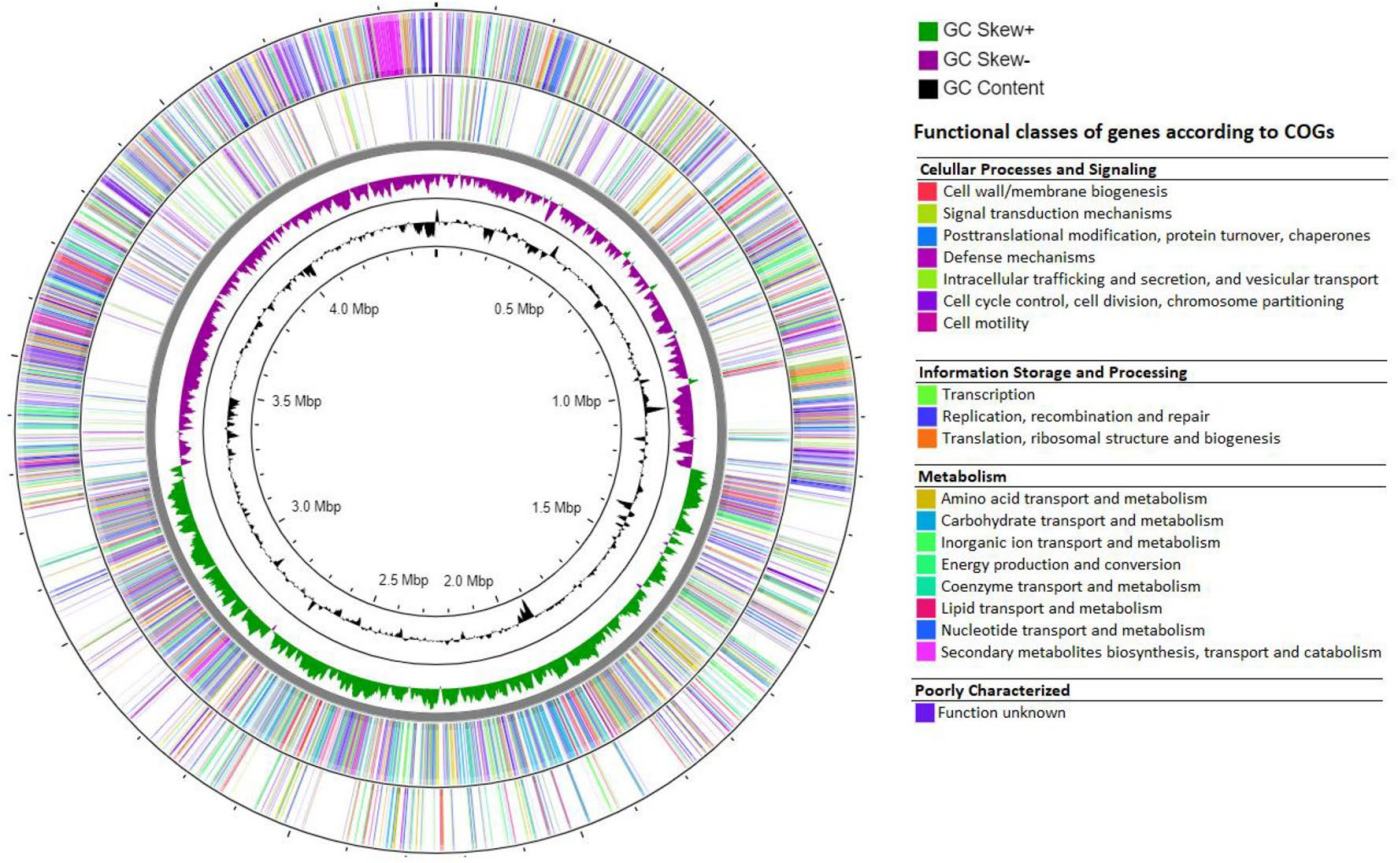
Circular analysis and visualization of *V. salarius* 19.PP.SC1.6 genome. From the outside to the inside, the first and second circle represent genes with COG annotation. Circles 3 (green and purple) and 4 (black) show GC skew and GC content as the deviation from the average for the complete genome.

The functional mapping of COG provides more insight into the analysis of CDSs (Fig. 3). *V. salarius* 19.PP.SC1.6 is found to possess 21 out of the 25 COG categories, with the absence of four specific categories: Y (related to nuclear structure), Z (related to the cytoskeleton), A (associated with RNA processing and modification), and R (associated with the prediction of general function only). The annotated *V. salarius* 19.PP.SC1.6 genome includes 266 pathways and 75 KEGG modules, as determined by the KEGG Reconstructor results.

### Pan genome analysis

**Figure 4:**
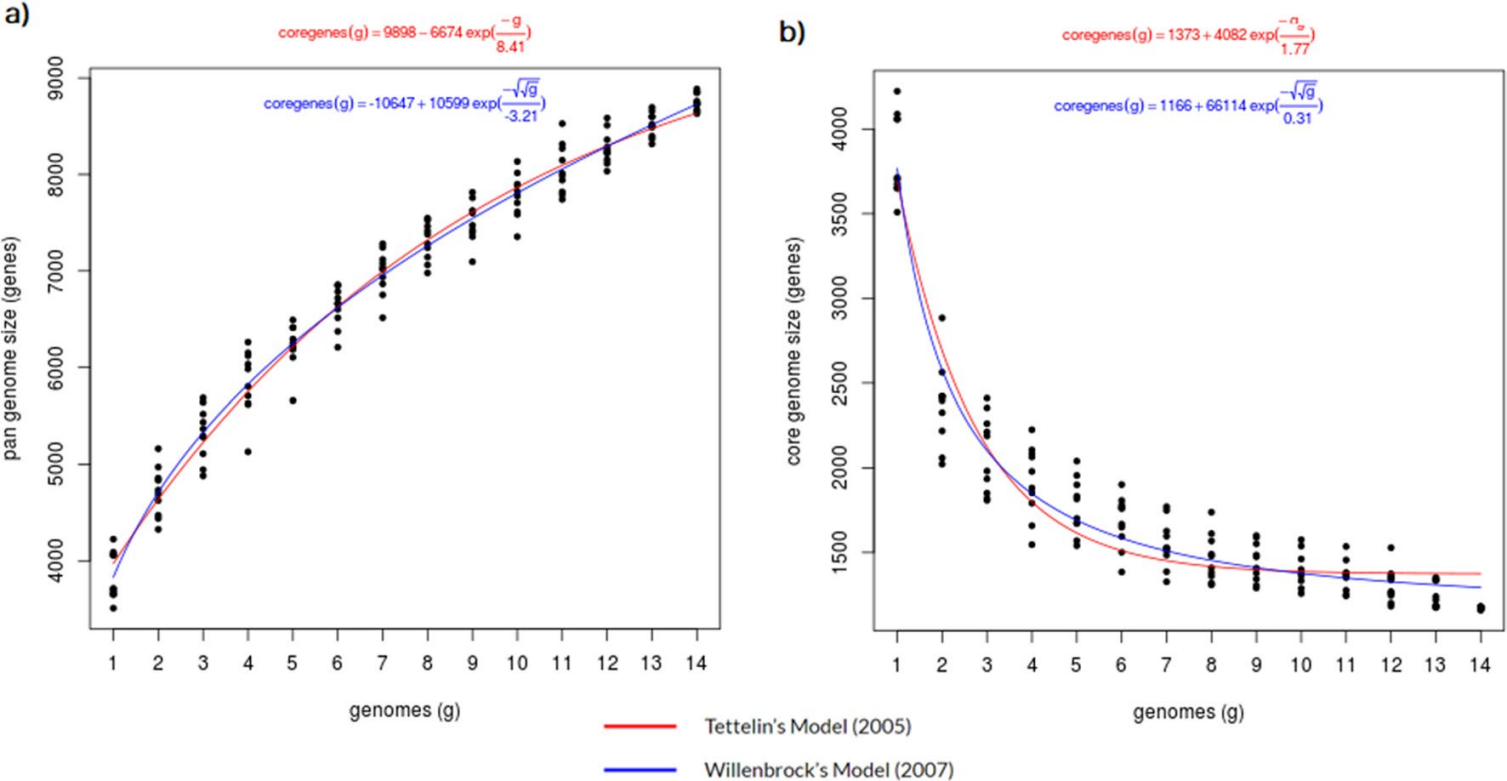
Pan- and core-genome estimation curves. (**a**) The size of the pan-genome increases for every included genome indicating an open pan-genome. (**b**) Core-genome size decreases with more genomes included in analysis.

The 14 *Virgibacillus* genomes (Supplementary Table S2) collectively exhibit an estimated pan genome size of 11,345 gene clusters. Within this genomic repertoire, 1136 genes constitute the core genome, 1596 genes make up the soft-core genome, 2295 genes form the shell genome, and a substantial 7463 genes constitute the cloud genome. Remarkably, the *Virgibacillus* core genome represents only 10.01% (1136 out of 11,345) of the entire pangenome. This relatively low proportion of the core genome suggests that *Virgibacillus* exhibits characteristics of an "open pangenome"^50^. Figure 4 illustrates the estimation curve for core genome size, which closely aligns with the size of the *Virgibacillus* genus pangenome, consistent with the concept of an open pan genome previously proposed by researchers^51, 52^.

Moreover, the determination of the type of pangenome is guided by calculating the value on the pangenome size curve derived from computational results with GET HOMOLOGUES (Fig. 4a). In the case of an open pangenome, a significant portion of its genes is unique and exclusive to specific species. A genus characterized by an open pan genome implies that the species within it possess adaptability to diverse ecological niches^53, 54^. This evidence lends support to the hypothesis that *V. salarius* 19.PP.SC1.6 may potentially harbor novel genes or produce unique secondary metabolites as an adaptation to its niche in the North Java region. Consequently, there is a compelling incentive to delve further into the exploration of the *V. salarius* 19.PP.SC1.6 genome.

### Prediction of genomic islands

**Figure 5:**
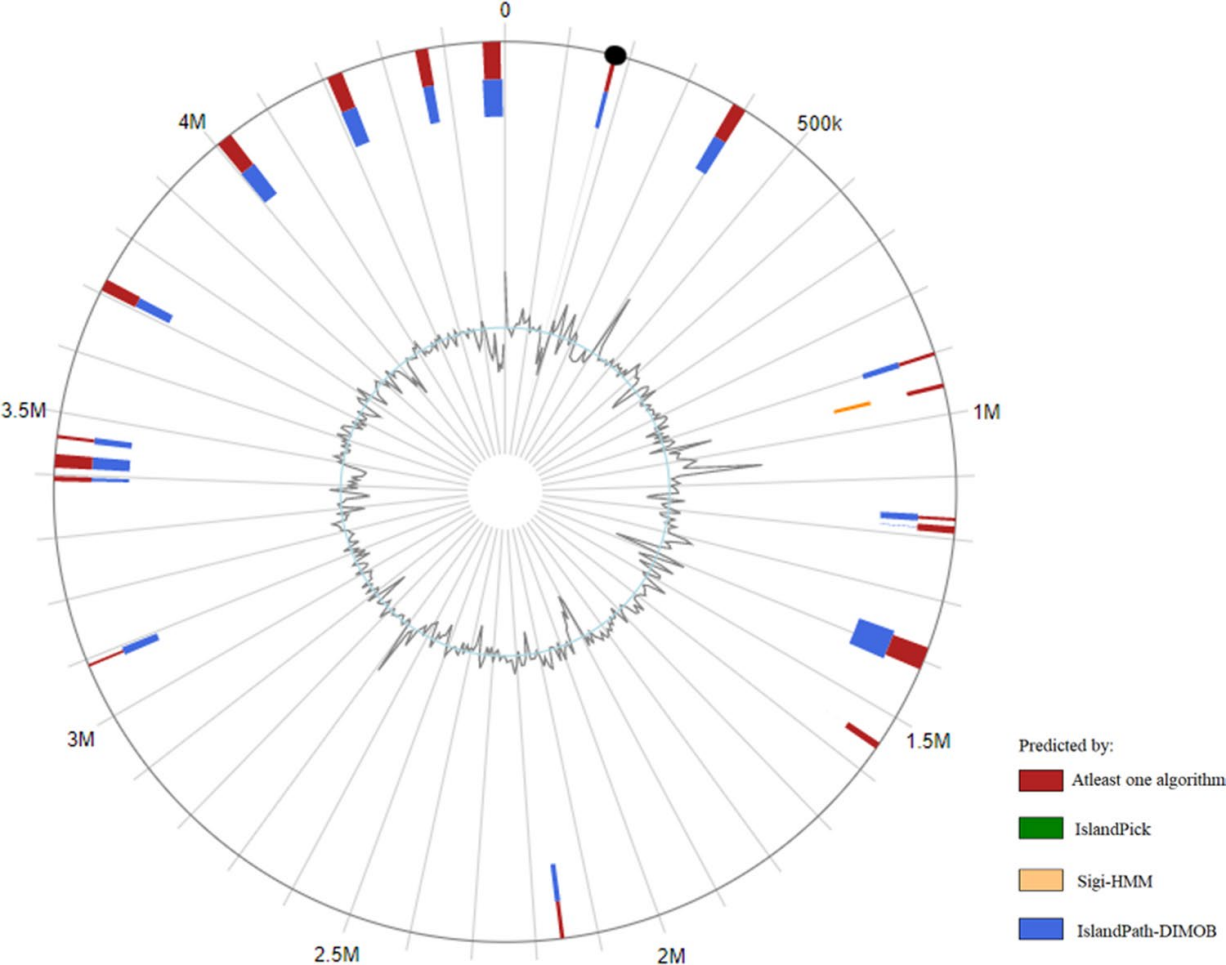
Visualization of Genomic Island detected in the *V. salarius* 19.PP.SC1.6 Genome using IslandViewer 4.0. The genome of *V. salarius* 19.PP.SC1.6 contains a total of 18 genomic islands.

The prediction of genomic islands using IslandViewer version 4.0 revealed the presence of a total of 18 genomic islands within the genome of *V. salarius* 19.PP.SC1.6 (Fig. 5). These genomic islands exhibit varying lengths, ranging from 4.385 to 36.931 kb, and each island contains between 4 and 41 genes. Notably, some of the genomic islands in *V. salarius* 19.PP.SC1.6 have relatively smaller sizes, ranging from 4 to 8 kb, in contrast to the average genomic island size, which typically falls between 10 and 200 kb and is commonly observed in other bacteria^55^.

Within the genomic island of *V. salarius* 19.PP.SC1.6, a total of 355 genes were identified. Significantly, 44.51% of these genes (158 out of 355) were not found in the COG database, indicating the presence of novel genes that have not been previously characterized. Additionally, 15.21% of the genes (54 out of 355) were classified as having an unknown function (category S). Furthermore, a substantial portion, approximately 59.71% of the total genomic island genes (212 out of 355), remain functionally uncharacterized. Among the remaining genes detected by the COG database, the dominant category observed was category L, which pertains to replication, recombination, and repair processes^55^.

The functions of other genes within the genomic island remain unknown, providing an opportunity for further research to uncover the specific types of genomic islands present in *V. salarius* 19.PP.SC1.6. These islands could potentially encompass various categories, including pathogenicity islands, resistance islands, metabolic islands, and symbiosis islands, among others, warranting further investigation.

### Prediction of secondary metabolite BGCs

We conducted a secondary metabolite prediction analysis using antiSMASH 6.0 to identify potential secondary metabolite gene clusters in *V. salarius* 19.PP.SC1.6. Table 3 provides information on the secondary metabolite gene clusters identified in the genome, with a total of seven clusters containing 3 to 30 genes. In aggregate, these seven gene clusters encompass a total of 80 genes.

**Table 3:** *V. salarius* 19.PP.SC1.6 secondary metabolite gene clusters from antiSMASH analysis results showed the presence of seven gene clusters with cluster lengths ranging from 2190 to 84,764 bp with a total of 80 genes.

Cluster	Start	Stop	Length (bp)	KCBLAST* result	Similarity (%)	Number of genes
Terpene	671,094	691,138	20,044	–	–	6
Terpene	2,533,865	2,555,214	21,349	–	–	7
NRPS	2,789,711	2,833,924	44,213	Bacillibactin	46	4
PKS (type III)	3,235,646	3,275,709	40,063	–	–	15
Ectoine	3,905,372	3,907,562	2,190	Ectoine	75	3
PKS (type III)	3,915,988	3,956,238	40,250	–	–	12
NRPS	4,367,900	4,452,664	84,764	Locillomycin	64	30

*KCBLAST (KnownCluster BLAST) is a part of the antiSMASH pipeline that aligns the query with clusters from the MiBIG database.

The first secondary metabolite gene cluster falls under the polyketide synthase (PKS) type III category and comprises two main genes: naringenin-chalcone synthase (NCS) and hydroxymethylglutaryl-CoA synthase (HMGCS). These enzymes play pivotal roles in the biosynthesis of naringenin chalcone and (S)-3-hydroxy-3-methylglutaryl-CoA (HMG-CoA)^56, 57^. Within *V. salarius* 19.PP.SC1.6, the terpene gene cluster includes the enzymes farnesyl-diphosphate farnesyltransferase (FDFT1) and squalene-hopene cyclase (SHC). These enzymes are responsible for generating sesquiterpenoid and triterpenoid derivatives^58, 59^.

Non-ribosomal peptides (NRPs) represent secondary metabolites known for their antibacterial activity. NRP 1 in the V. salarius 19.PP.SC1.6 genome exhibits a predicted similarity of 0.542 to bacillibactin, with an antibacterial confidence of 0.8122 against *Klebsiella oxytoca*. Meanwhile, NRP 2 is anticipated to have the capacity to produce new compounds akin to lolicillomycin and 51W pyoverdine. Furthermore, the ectoine gene cluster in *V. salarius* 19.PP.SC1.6 comprises a complete set of genes, consisting of *ectABC*, which will be further examined for its ability to produce intact ectoine products.

### Characterization and comparison of ectoine biosynthetic gene cluster

According to antiSMASH results, three genes in the ectoine gene cluster are located on the *V. salarius* 19.PP.SC1.6 genome at bases 3,905,306–3,907,562 with a total length of 2190 bp. These three genes code l-ectoine synthase (*ectC*), diaminobutyrate-2-oxoglutarate transaminase (*ectB*), and L-2,4-diaminobutyric acid acetyltransferase (*ectA)*.

**Figure 6:**
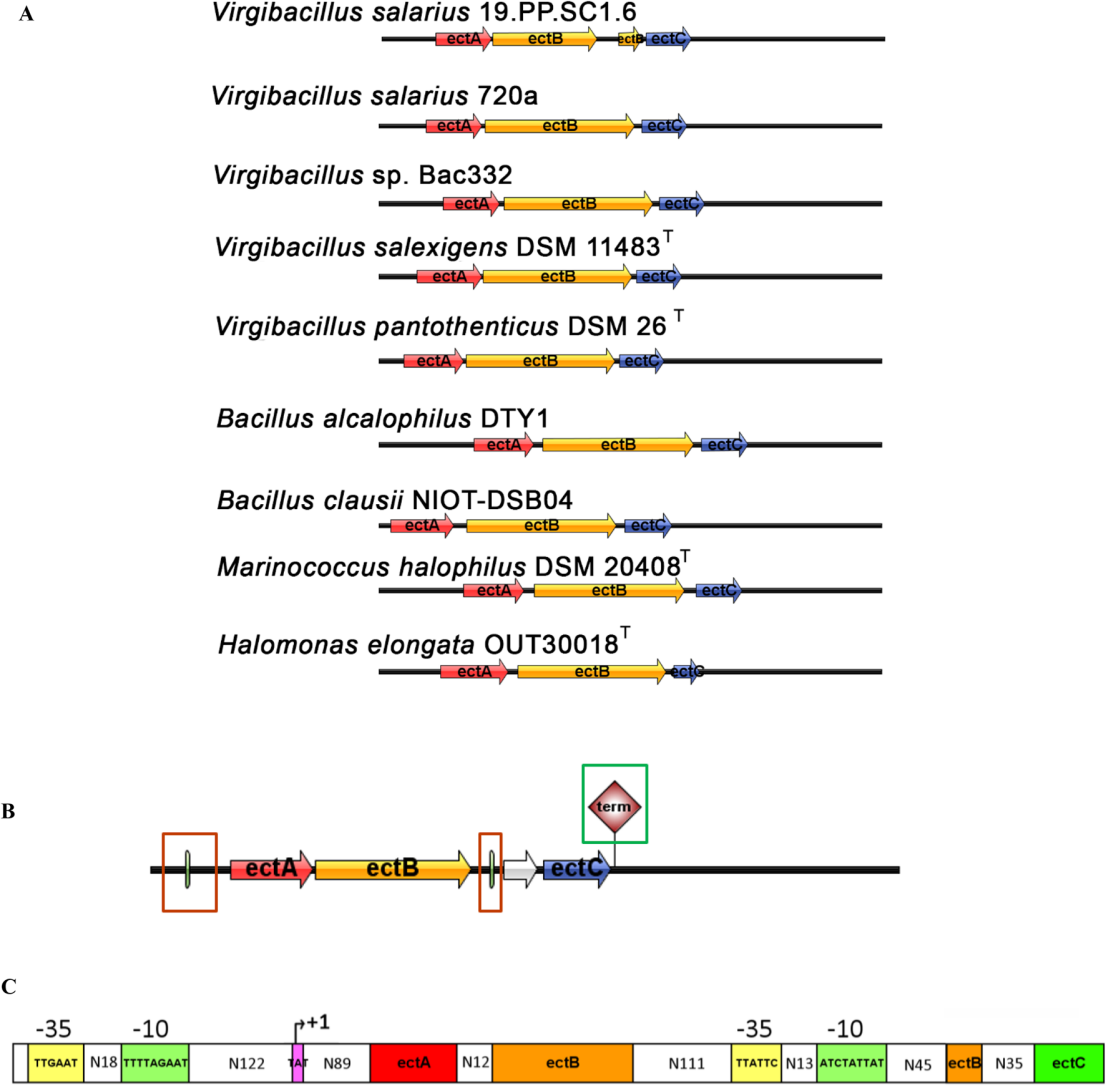
Ectoine gene cluster. (**A**) Comparative schematic structure of ectoine biosynthetic gene organization in *Virgibacillus salarius* and other ectoine producing bacteria. l-2,4-diaminobutyric acid N-γ-acetyltransferase genes (*ectA*) are red, diaminobutyric acid transaminase genes (*ectB*) are yellow, and ectoine synthase (*ectC*) are blue. (**B**) Ectoine gene cluster in *Virgibacillus salarius* from WGS result showed two operons comprised of *ectABC* genes with two promoter (red box) and one terminator (green box). (**C**) Putative structural features of *V. bacillus* ectoine biosynthetic gene cluster, consist of − 35 and − 10 boxes, with transcription start site (+ 1).

The complete sequence of ectoine-producing bacteria gene clusters, consisting of non-coding and coding regions, was predicted for the number of operons, number of genes, and gene length; the structure of the gene cluster was also visualized (Fig. 6A). Overall, ectoine gene clusters in all species have the same gene order, namely *ectA, ectB*, and *ectC*, with more or less the same gene size (*ectA* 479–557 bp; *ectB* 1265–1286 bp; *ectC* 332–398 bp). The ectoine biosynthetic gene cluster in *V. salexigens*, *V. panthotenthicus*, *B. alcalophilus*, *B. clausii*, *M. halophilus*, and *H. elongata* consists of one operon. However, there is a gap between *ectB* and *ectC* in the annotation results of the *V. salarius* 19.PP.SC1.6 gene cluster, resulting in the formation of two distinct operons. *H. elongata* had the largest gene cluster size, with a total of 2431 bp. Meanwhile, *V. pantothenticus* has the smallest ectoine gene cluster size, with a total of 2186 bp.

A total of 2190 bp of *V. salarius* 19.PP.SC1.6 ectoine gene cluster sequences were taken from the non-coding *ectA* upstream region to the non-coding *ectC* downstream region. Predictions using BPROM and FindTerm showed that the *V. salarius* 19.PP.SC1.6 ectoine gene cluster contains two promoters and one terminator; a visualization of the gene structure is shown in Fig. 6B. The first promoter is on the first operon, specifically the 239th base, with -10 boxes on the 224th base and -35 boxes on the 200th base (Fig. 6C) with predicted transcription factors marR, gcvA, and lrp. The other promoter was predicted at base 145 in the second operon, specifically in the non-coding downstream *ectB* region with − 10 boxes at positions 130 and − 35 boxes at positions 111 with crp as predicted transcription factor.

Bacterial transcription terminators control gene expression in two ways: intrinsic terminators that separate transcription complexes without the help of other factors (Rho-independent) and terminators that rely on RNA helicases called Rho (Rho-dependent). It is predicted that one putative Rho-independent terminator will be formed from the two operons formed in the *V. salarius* 19.PP.SC1.6 ectoine gene cluster, namely in the downstream non-coding region of *ectC*, starting from base 22 and until base 69.

The amino acid sequences of *ectABC V. salarius* 19.PP.SC1.6 and other ectoine-producing bacteria were aligned using MSA and compared for sequence identity and similarity. According to the findings, the *ectA* protein of *V. salarius* 19.PP.SC1.6 has 93.08% identity and 96.22% similarity to *V. salexigens*, with the highest predicted global similarity (BLOSUM62) of 0.93. The *ectB* gene from *V. salarius* 19.PP.SC1.6, like its *ectA* gene, has the highest identity and similarity to the *ectB* gene from *V. salexigens*, with similarity rate of 89.22% and 92.81%, respectively. Furthermore, BLOSUM 0.88. *ectC V. salarius* 19.PP.SC1.6 had the highest identity, similarity, and BLOSUM when aligned with *ectC V. salexigens*, with values of 97.65%, 99.21%, and 0.98, respectively.

**Figure 7:**
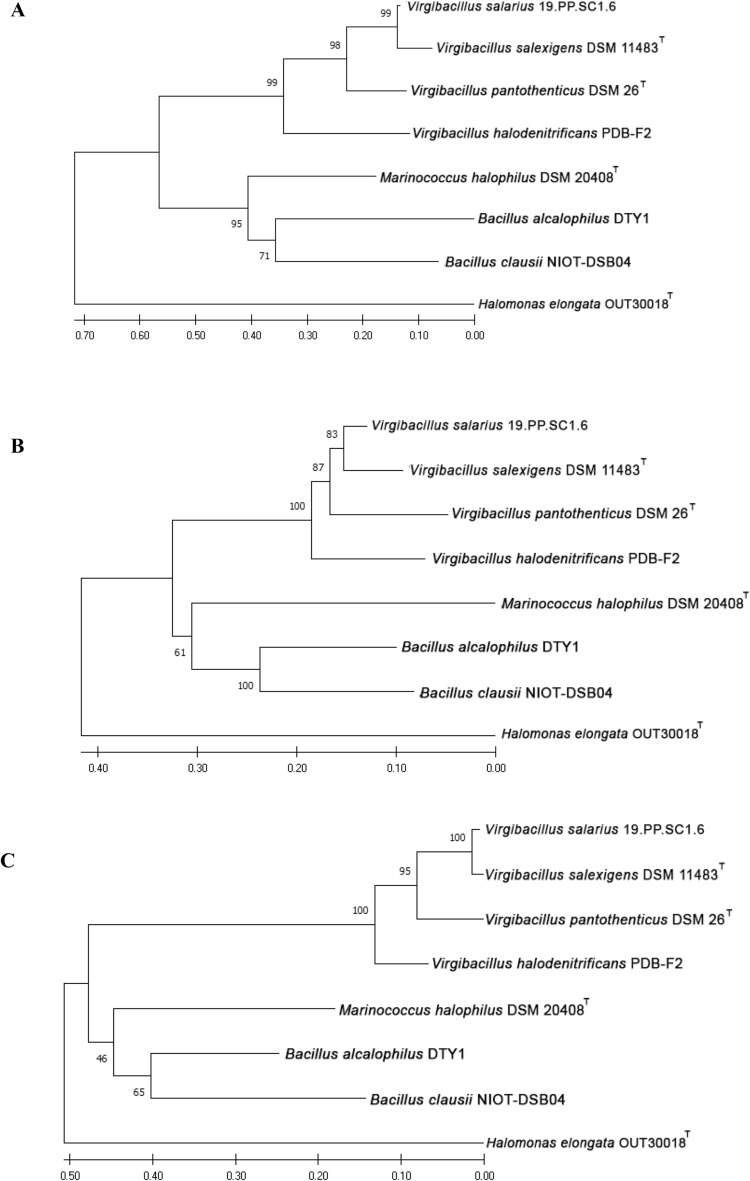
Phylogenetic analysis of *Virgibacillus salarius'* (**A**) *ectA*, (**B**) *ectB*, and (**C**) *ectC* and other ectoine-producing bacteria deposited in the NCBI Database. Sequences were aligned using MEGA 10 and phylogenetic trees were built using the neighbor-joining (NJ) method within the MEGA 10 software. *V. salarius' ectABC* amino acid sequences were closely related with *V. salexigens.*

After determining the sequences' similarity and identity, a Neighbor-Joining (NJ) phylogenetic tree was constructed (Fig. 7). *ectA*, *ectB*, and *ectC* of *V. salarius* 19.PP.SC1.6 were found to be the most closely related to *ectABC* of *V. salexigens* based on phylogenetic analysis. This is supported by the results of the previous sequences' similarity and identity, with *ectABC V. salarius*-*V. salexigens* having the highest percentage of similarity and identity. It was also known that the *Virgibacillus* clade and the *Marinococcus-Alkalihalobacillus* clade had formed, indicating that *ectABC* from *M. halophilus* had the highest similarity to the *ectA* genus *Alkalihalobacillus*, as well as the presence of outgroup *H. elongata.*

### Ectoine gene cluster isolation

The *ectABC* gene amplification results indicated that the *ectA* gene had a size of 480 bp, the *ectB* gene was 1245 bp, and the *ectC* gene was 387 bp (Supplementary Fig. S1). These findings align with the in silico annotation results, confirming the successful isolation of the *ectABC* gene cluster of *V. salarius* 19.PP.SC1.6. To obtain the full-length sequence of these three genes, we performed pairwise alignment between the whole genome sequencing (WGS) sequence of *V. salarius* 19.PP.SC1.6, which had been annotated in silico, and the plasmid sequencing containing the *ectA*, *ectB*, and *ectC* sequences from *V. salarius* 19.PP.SC1.6 using the EMBOSS-Water tool.

The pairwise sequence alignment (PSA) between the plasmid sequencing and WGS *ectA* sequences revealed 100% similarity and sequence identity, spanning 480 bp of the *ectA* sequences. PSA conducted between the *ectB* WGS sequences and the *ectB* plasmid sequence exhibited 99.8% similarity and identity with a 0.1% sequence gap. Notably, two nucleotide base differences and one base substitution were observed in the *ectB* WGS sequence, leading to a gap in the sequence alignment results. These discrepancies between the WGS results and the plasmid sequence caused a frameshift in the *ectB* sequence, resulting in a stop codon occurring in the middle of the *ectB* gene. PSA performed on the *ectC* plasmid sequence against the *V. salarius* 19.PP.SC1.6 *ectC* WGS result revealed 99.7% identity and similarity with no sequence gaps. There was one nucleotide-based difference between the *ectC* plasmid sequence and the *ectC* WGS sequences, leading to a distinct amino acid sequence. In the *ectC* WGS sequence, Proline (P) was found, while Leucine (L) was present in the *ectC* plasmid sequence. These variations in sequence between the WGS and plasmid results may be attributed to differences in sample specificity during sequencing. The WGS approach employed samples from the entire *V. salarius* 19.PP.SC1.6 genome, resulting in a much larger number of genes and nucleotide bases being read. In contrast, plasmid sequencing utilized specific primers (T7 and SP6 promoters), resulting in a smaller and more specific set of bases being read, as only the *ectABC* gene was amplified.

**Figure 8:**
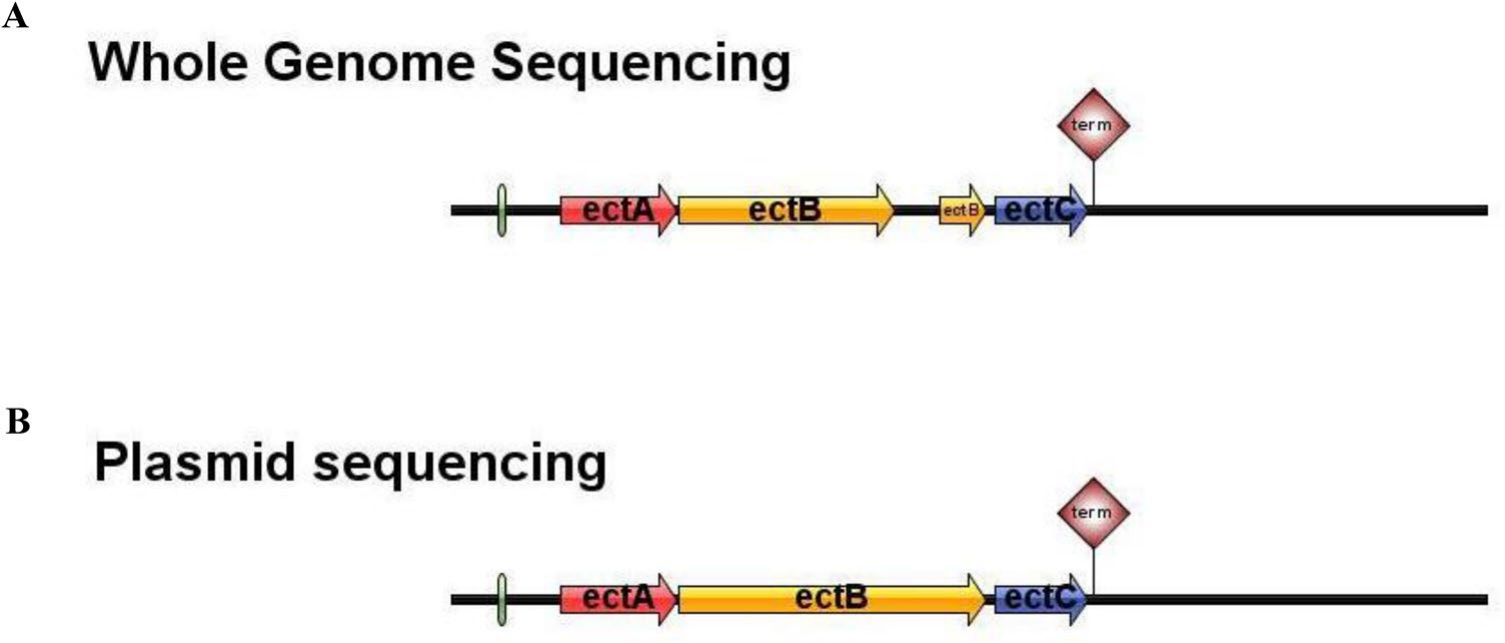
Comparative schematic representation of the *V. salarius* ectoine gene cluster structure based on (**A**) WGS and (**B**) plasmid sequencing results. Plasmid sequencing revealed that ectoine gene cluster forms one operon consisting of *ectABC* gene with the promoter shown in the green box and terminator shown in the red triangle.

To further confirm the *ectB* and *ectC* sequences, we conducted pairwise sequence alignments between *V. salarius*' *ectB* and *ectC* sequences and those of closely related species, specifically *V. salexigens ectB* and *ectC* (Supplementary Figs. S2–S3). These results were then employed for visualizing the ectoine gene cluster between the plasmid and WGS sequences. Initially, in silico analysis suggested that the *V. salarius* 19.PP.SC1.6 ectoine gene cluster consisted of two operons, each with two promoters and one terminator (Fig. 8A). However, after isolating and sequencing *ectA*, *ectB*, and *ectC*, it was revealed that the *V. salarius* 19.PP.SC1.6 ectoine gene cluster comprised only one operon, with one promoter and one terminator (Fig. 8B).

### Ectoine production

**Figure 9:**
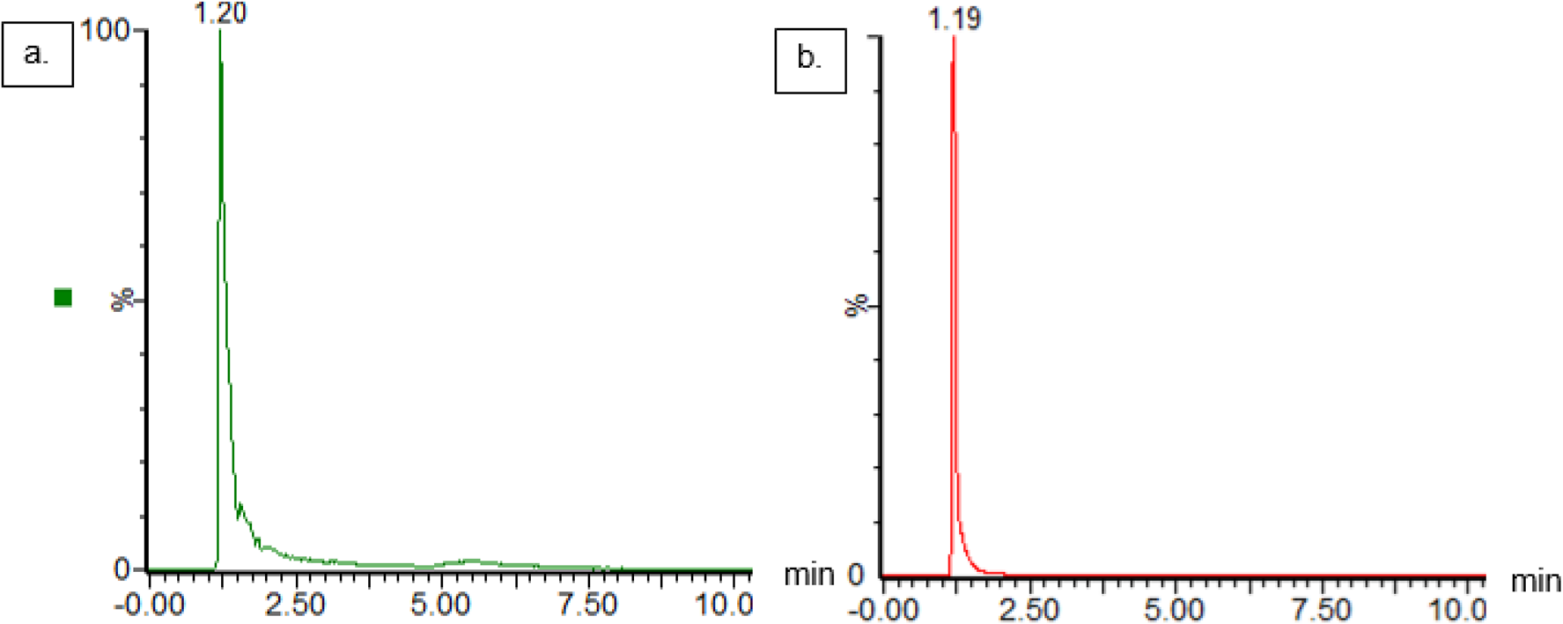
Ectoine biosynthesis analysis of *Virgibacillus salarius.* UPLC-MRM-MS/MS map (**a**) The UPLC map shows spectra of intracellular ectoine extracted from *V. salarius*. (**b**) The UPLC map of the authentic standard of ectoine. The number shown in the map indicates that intracellular ectoine from *V. salarius* has high similarity in structure as compared to pure ectoine used as control.

The results of the whole genome sequencing (WGS) and the characterization of the *V. salarius* 19.PP.SC1.6 ectoine biosynthetic gene cluster were further investigated to determine if the gene cluster functions as a whole to synthesize ectoine. The presence of ectoine in *V. salarius* 19.PP.SC1.6 incubated in Marine Zobell medium with 15% NaCl was confirmed using UPLC-MS/MS operated in MRM mode, as shown in the chromatograms (Fig. 9). This indicates that *V. salarius* 19.PP.SC1.6 possesses intact ectoine gene clusters and is capable of producing ectoine as compatible solutes, which act as osmotic protective agents for bacterial cells. Further quantification of ectoine using our validated analytical method (Supplementary Table S1) showed that cells incubated for 30, 45, and 48 h in 10% NaCl produced 0.23, 0.46, and 0.68 mg of ectoine per liter of medium culture, respectively. This preliminary study revealed that the ectoine yield from *V. salarius* 19.PP.SC1.6 is lower compared to several previously reported or commercial ectoine-producing bacteria (1170–14,860 mg/L)^60–62^. However, previously reported yields are the result of engineering and optimization process and cannot be compared directly. Efforts such as growth optimization and other molecular approaches, including metabolic pathway optimization, might be employed to increase the yield of ectoine from *V. salarius* 19.PP.SC1.6.

## Conclusion

Genome characteristics such as gene annotation, number of genomic islands, pan genome types, and number of secondary metabolite gene clusters from *V. salarius* 19.PP.SC1.6 have been obtained. Terpene groups, NRPS, PKS (type III), and ectoine are four of the seven clusters of secondary metabolite-producing genes found. The *V. salarius* 19.PP.SC1.6 ectoine biosynthetic gene cluster, which includes the *ectABC* gene, has been characterized, and *V. salarius* ' ability to produce ectoine has been identified. Overall, our study has uncovered several potential metabolites from *V. salarius* 19.PP.SC1.6, and this opens the opportunity for further utilization.

### Supplementary Information

Supplementary Information 1.

Supplementary Information 2.

## Data Availability

The genome sequence of the *V. salarius* 19.PP.SC1.6 has been deposited in GenBank/EMBL/DDBJ under the accession number GCF_027941815.1.
