# Supplementary material for: Biosynthetic gene cluster profiling from North Java Sea Virgibacillus salarius reveals hidden potential metabolites
Source: Sci Rep. 2023 Nov 6;13:19273. doi: 10.1038/s41598-023-44603-8 (PMC10630419; doi:10.1038/s41598-023-44603-8)
Supplement: Supplementary file 1 — Supplementary Information 1. [file 41598_2023_44603_MOESM1_ESM.pdf]

Supplementary Information 1

Supplementary Table S1. Validation of linearity and sensitivity of ectoine analysis using LC-MS

**Parameter**	**Result**
Retention time (min, n=3)	1.21
Linear regression	y= 128281x – 4623.6
Correlation coefficient (R^2)	0.9999
Range of linear concentration (µg/mL)	0.48-31.25
Limit of detection(µg/mL)	0.0048
Limit of quantification (µg/mL)	0.0145

Supplementary Table S2. The genome of various *Virgibacillus* species

Species	Accession Number
V.pantothenticus_DSM26	GCF_018075365
V.necropolis_LMG19488	GCF_002224365
V.phasianinus_LM2416	GCF_002216775
V.halodenitrificans_PDBF2	GCF_001878675
V.dokdonensis_21D	GCF_002849835
V.halodenitrificans_Bac324	GCF_003667805
V.halodenitrificans_ASH15	GCF_021391295
V.sp_6R	GCF_001895305
V.sp_Bac332	GCF_003667785
V.sp_Bac330	GCF_003667845
V.sp_NKC1916	GCF_021560035
V.sp_SK37	GCF_000725285

V.sp_MSP41	GCF_010092505
V.salarius_720a	GCF_018139605
V.sp_AGTR	GCF_016919725

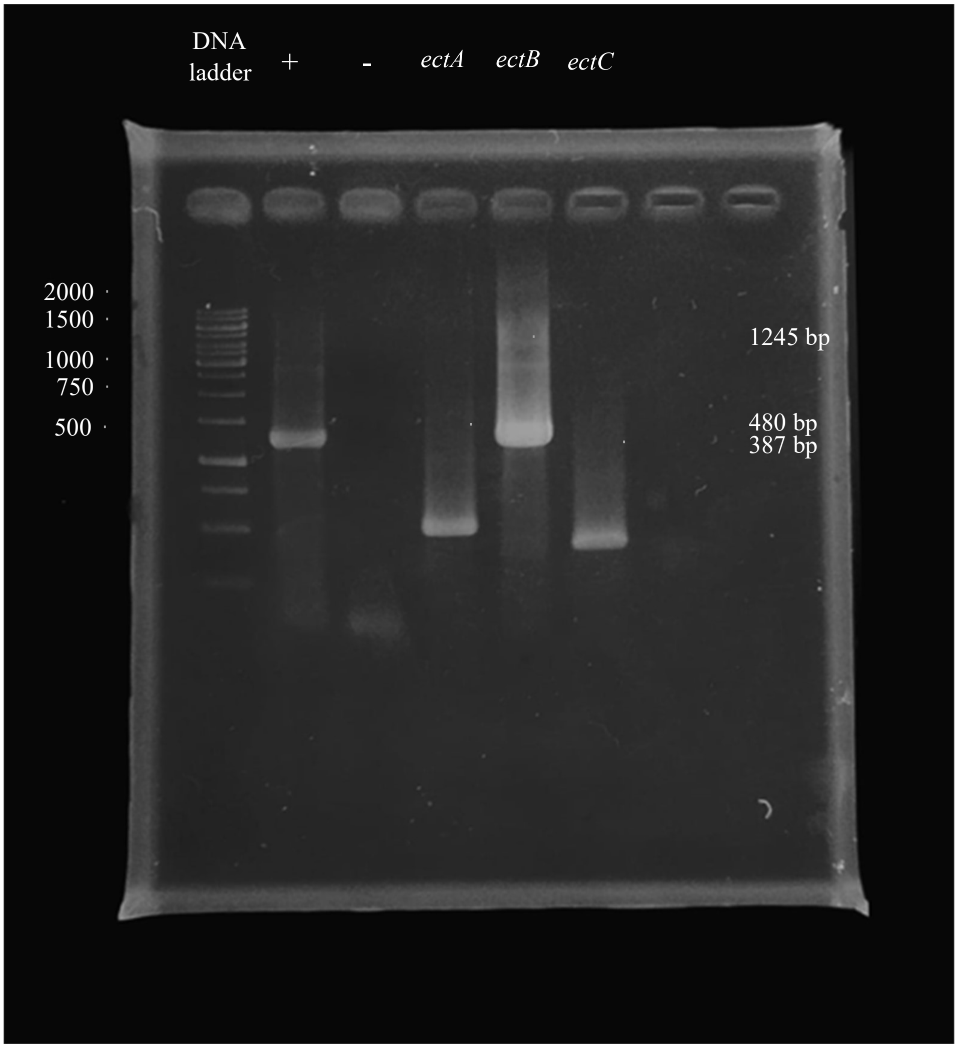

Supplementary Figure S1. Gel electrophoresis of the *ectA, ectB, ectC* gene from *V. salarius*. Lane 1 and 2 consist of positive and negative control, meanwhile lane 3-5 shows *ectA* (480 bp), *ectB* (1245 bp), and *ectC* (387 bp) genes.

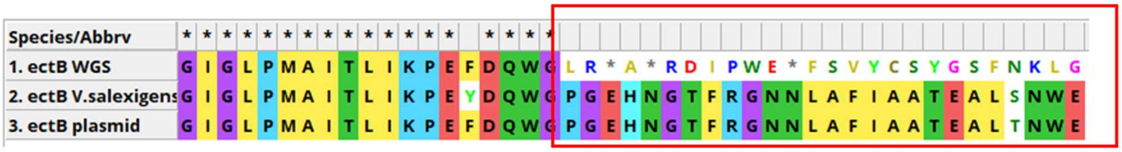

Supplementary Figure S2. Multiple sequence alignment of EctB plasmid and EctB WGS *V. salarius* with EctB *V. salexigens* using MEGA-X software.

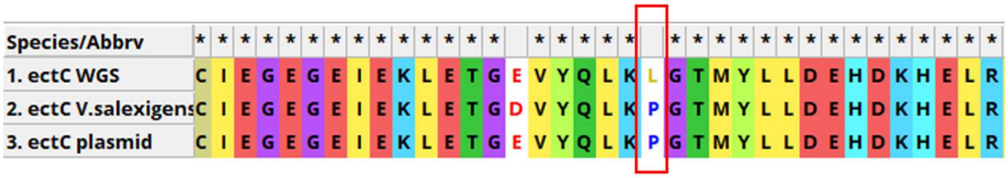

Supplementary Figure S3. Multiple sequence alignment of EctC plasmid and EctC WGS *V. salarius* with EctC *V. salexigens* using MEGA-X software.
